# Primary stability and PES/WES evaluation for immediate implants in the aesthetic zone: a pilot clinical double-blind randomized study

**DOI:** 10.1038/s41598-021-99218-8

**Published:** 2021-10-08

**Authors:** Arturo Sanchez-Perez, Ana I. Nicolas-Silvente, Carmen Sanchez-Matas, Silvia Molina-García, Carlos Navarro-Cuellar, Georgios E. Romanos

**Affiliations:** 1 Department of Periodontology, Medicine and Dentistry Faculty, CEIR Campus Mare Nostrum, University of Murcia, Murcia, Spain; 2 School of Dentistry, CEIR Campus Mare Nostrum, University of Murcia, Murcia, Spain; 3 Virgen del Rocio Hospital, 41013 Sevilla, Spain; 4 Murcia, Spain; 5 Department of Surgery, Complutense University, Madrid, Spain; 6 Department of Periodontology, School of Dental Medicine, Stony Brook University, Stony Brook, NY 11794-8712 USA

**Keywords:** Biological techniques, Anatomy, Health care, Risk factors

## Abstract

The use of immediate implants in the aesthetic area is a technique widely used in modern implantology. The characteristics of the patient, the implant, and the surgical procedure used may influence the final results. The aim was to assess whether the implant design affects primary (P.S.) and secondary stability (S.S.), bone level (B.L.), and PES/WES evaluation. Twenty implants with two different designs (n = 10) were immediately placed and randomly located in the upper anterior maxilla with no grafting material. Implant-Stability-Quotient (ISQ), B.L., and Pink-Esthetic-Score/White-Esthetic-Score (PES/WES) were evaluated. Shapiro–Wilk normality test was performed to determine the sample normality, as the data did not follow a normal distribution, the Wilcoxon-Mann–Whitney test was applied (*p* < 0.05). ISQ was determined at placement (PS): control 59.1 (C.I.54.8–63.3); experimental 62.2(C.I.60.1–64.2) and three months after placement (SS): control 62.2.1 (C.I.53.3–71.0); experimental 67.2(C.I.65.8–68.5). The BL was measured at three months after placement: control 0.38 mm (C.I.− 0.06 to  + 0.83); experimental 0.76 mm (C.I.0.33–1.19) and at 12 months post-loading: control 0.07 mm (C.I.− 0.50–0.65); experimental 0.90 mm (C.I.0.38–1.42). PES/WES values were evaluated for the control group: 15 (C.I.12.68–17.32), and for the experimental group 15.20 (C.I.11.99–18.41). No significant differences were shown between both implant designs. A good grade of osseointegration and primary/secondary stability was achieved, as well as proper maintenance of crestal bone and adequate PES/WES scores. The criteria for selection for the ideal patient for immediate implant placement is essential.

**ClinicalTrials Protocol ID**: NCT04343833.

## Introduction

The first manuscript about immediate implants was published in 1976 by Schulte and Heimke; they evaluated implants with aluminum oxide surface and a stepped morphology^1^. Several years later, Lazzara in 1989 disseminated this new technique^2^. Since then, the insertion of immediate implants has become a widely used technique.

This technique has several advantages, among them a lower treatment time, a few numbers of surgeries needed, the possibility of placing the implant in an axial position, but, above all, the positive psychological impact on the patient^3,4^. On the other hand, some risks have also been documented, such as a higher percentage of failures^5–11^.

To overcome these inconveniences, new strategies have been developed, such as the use of flapless techniques to minimize bone reabsorption^12,13^, the improvement of aesthetics through soft tissue management, both with periodontal surgeries^14^ or with the use of temporary prostheses which provides adequate support of the supracrestal complex and proper sealing of the socket^15^, and also with the support of bone grafting and connective tissue techniques, although without conclusive results^16,17^.

At the industry level, manufacturers have increased their efforts by developing new macro- and micro-designs and new surfaces that increase both primary stability and the osseointegration process^18^. Also, a large number of studies have appeared evaluating in a standardized way, different conditions, designs, surgical protocols, and have reached high clinical relevance.

The objective of this study was to determine whether a new macro- and micro-geometric design en boasted better primary stability and better aesthetic results in immediate implants placed in the aesthetic zone when compared to a conventional design implant.

## Materials and methods

### Sample

#### Sample description

A total of 20 patients, distributed in two groups with ten patient each (experimental and control) were included in this study. All patients signed a double informed consent, one for implant placement and one for their participation in the study. The study follows the Declaration of Helsinki recommendations^19^. The Ethics Committee of the University of Murcia approved the study protocol with ID: 2076/2018, and was registered at ClinicalTrials with ID: NCT04343833 on 13/04/2020. All patients were treated in the Dental School, University of Murcia, as they requested for treatment from 02/10/2017 to 19/09/2019. The preselection was performed by a dentist who was unaware of the subsequent allocation to the treatment groups (AINS).

#### Inclusion criteria

All patients were older than 18 years old who present a vertical fracture in an upper incisor that can not be treated conservatively and ended in the extraction, and the treatment needed is a unitary implant. Both males and females were included in the sample. Good systemic health status (ASA I or II), an oral hygiene index of < 2 (Löe and Silness), a minimum of 2 mm of attached mucosa, a minimum of 8 mm of vertical bone, and a minimum of 7 mm of bucco-lingual bone.

#### Exclusion criteria

Traumatic or complicated incisor extraction, pregnant or women in the lactation period, the use of any medication that contraindicates implant treatment, a history of alcohol or drug abuse, a requirement for guided bone regeneration procedures or soft tissues augmentation procedures, and failure to comply with the study protocol.

### Implants

All implants were developed by the same manufacturer and were approved by the regulations of the European Certificate. The traceability was guaranteed by Q.R. Genetic Codes® (Ticare, Mozo Grau, Valladolid, Spain).

In the control group (Group A), patients were treated with conventional implants Inhex Ticare Standard (Mozo Grau, Ticare, Valladolid, Spain). The implants presented a surface treated with Reabsorbable Blast Media (RBM), conical macro-design with non-aggressive threads, internal connection, and platform switching. On the implant shoulder, the beveled design of the platform was characterized by a rounded shape of 45°.

**Figure 1:**
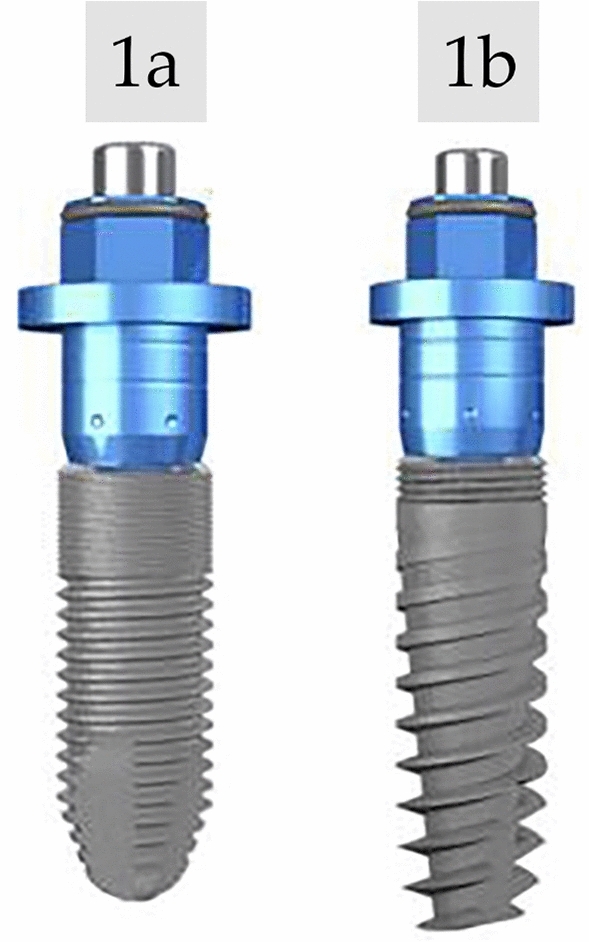
Implant design (**a**) Design for the control group (Inhex Ticare Standard, Mozo Grau, Ticare, Valladolid, Spain); (**b**) Design for the experimental group (Inhex Quattro Ticare, Mozo Grau, Ticare, Valladolid, Spain).

In the experimental group (Group B), patients were treated with Implants Inhex Quattro Ticare (Mozo Grau, Ticare, Valladolid, Spain). The implants also presented a surface treated with RBM, conical macro-design with expanded micro threads, internal connection, and platform switching. The implant shoulder showed the same beveled design than group A (Fig. 1).

## Study Protocol

**Figure 2:**
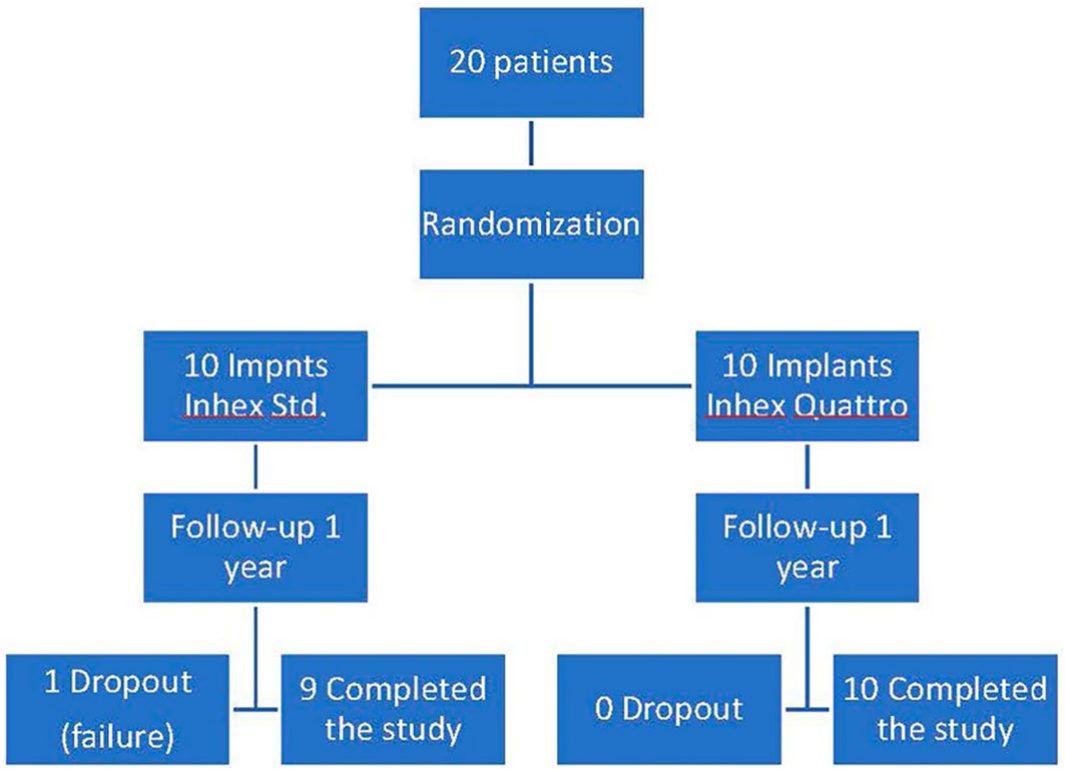
Protocol for randomized distribution of the sample. Distribution of the sample and the final number of dropouts and samples with completion of the study.

A total of 20 patients were randomly distributed into two groups (Fig. 2) following the randomization application https://www.random.org/sequences/:

- Group A (control) (n = 10) (Inhex Ticare Standard, Mozo Grau, Ticare, Valladolid, Spain)
- Group B (experimental) (n = 10) (Inhex Quattro Ticare, Mozo Grau, Ticare, Valladolid, Spain)

The distribution was kept in a numbered envelope until the time of the intervention. Each patient was numbered consecutively, as admitted in the study, and the corresponding envelope opened at the time of surgery.

**Figure 3:**
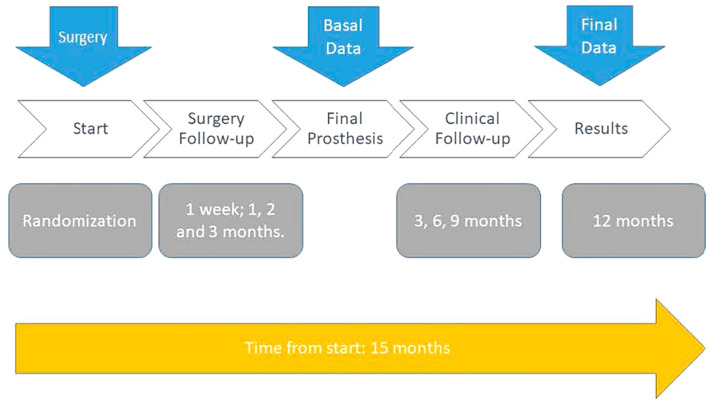
Scheme of the follow-up protocol for treated patients.

All patients were treated by the same surgeon (ASP), who was informed of the assigned group just before surgery. At the time of operation, the tooth was atraumatically extracted, and the implant was immediately placed following the surgical protocol recommended by the manufacturer. A temporary prosthesis was made without occlusal contact. The protocol followed by all patients is schematized in Fig. 3.

Patients were followed-up at one-week, one-month, two-months, and three-months post-surgery. Once the osseointegration process was reached, the implant was loaded with the definitive prosthesis, and the patient was monitored every three months until one-year post-loading, assuming a total follow-up time of 15 months. Controls were performed at the University Dental Clinic Of the University of Murcia. The data were stored on a spreadsheet and kept anonymous.

### Implant stability

The implant stability was evaluated in two different moments: At the time of the implant insertion (Primary Stability) and three months after surgery, just before placing the definitive prosthesis (Secondary Stability). This implant stability was measured through the Resonance Frequency Analysis System (Osstell®). The response obtained is measured in a value called implant stability quotient (ISQ) following a scale from 0 to 100. The higher the ISQ value, the greater implant stability^20^.

### Clinical images

Clinical photographs were obtained using a Nikon D70 camera, equipped with a 105 mm Sigma lens and a Meike MK-14EXT annular flash at a 1:1 magnification and the following parameters: 1/60, f29, and ISO 400. The same clinician took all the images.

### Pink esthetic score/white esthetic score (PES/WES)

A trained blind evaluator measured the images obtained during patient follow-up. The evaluator made the measurements through the digital photographs projected on a 200 × 200 cm wall display. All photographs were examined in the same room, with the same light and environmental conditions to avoid variations in the perception of the images.

#### Parameters evaluated by PES

**Figure 4:**
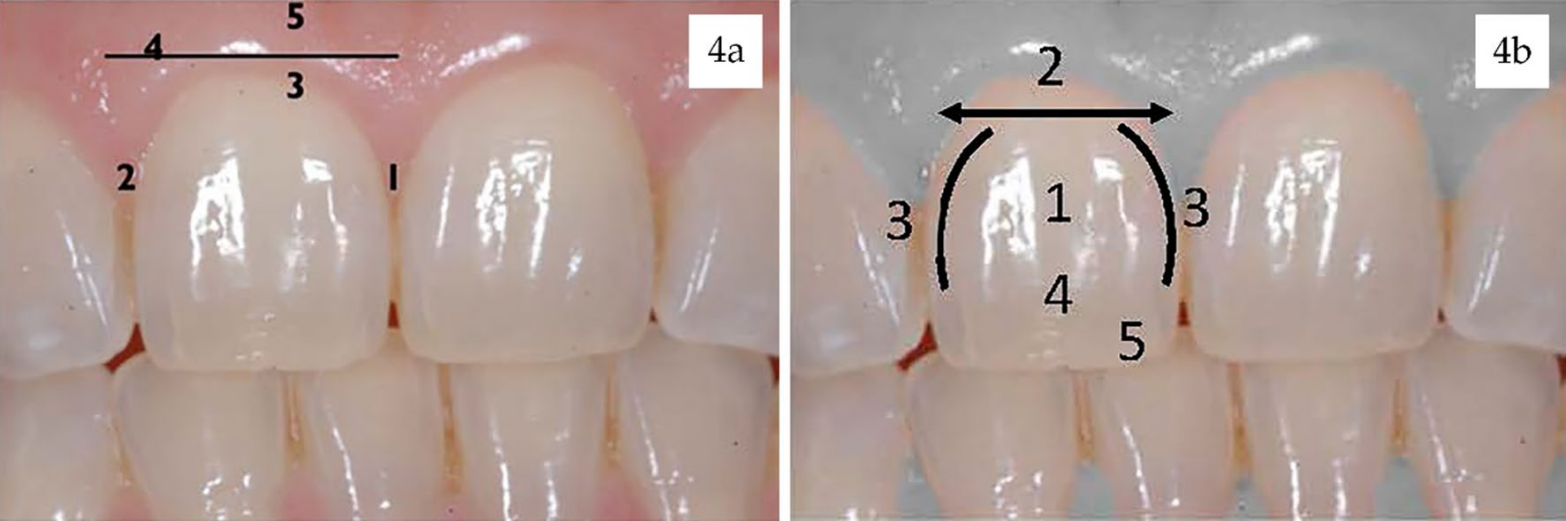
PES/WES Evaluation. (**a**) Parameters evaluated by WES and (**b**) parameters evaluated by PES.

The parameters evaluated by this index are (1) mesial papilla, (2) distal papilla, (3) curvature of facial mucosa, (4) level of facial mucosa, and (5) root convexity/soft tissue color and texture (Fig. 4). Each parameter range between 0 and 2. A maximum score of 10 can be obtained, considering acceptable from 6^21^.

#### Parameters evaluated by WES

The WES focuses specifically on the visible part of the restoration and in base on the following five parameters: (1) tooth form, (2) tooth volume/outline, (3) color (hue and value), (4) Surface texture, and (5) translucency (Fig. 4). Each parameter is evaluated by comparison with the adjacent teeth, giving a value ranged from 0 to 2. A total score of 10 can be obtained, considering acceptable from 6^21^.

Therefore, in the PES/WES evaluation, a maximum score of 20 can be achieved and represents the optimal state for both soft and hard tissues of the rehabilitated teeth compared with the adjacent teeth. The minimum acceptable PES/WES score is set to 12^21^. (Table 1).

**Table 1:** Detailed description of the parameters evaluated by the PES (a) and WES (b).

PES (a)			
Parameter	Absent	Incomplete	Complete
(1) Mesial papilla	0	1	2
(2) Distal papilla	0	1	2
	Major discrepancy	Minor discrepancy	No discrepancy
(3) Curvature of facial mucosa	0	1	2
(4) Level of facial mucosa	0	1	2
(5) Root convexity/soft tissue color and texture	0	1	2
Maximum total Modified PES score			10

WES (b)			
Parameter	Major discrepancy	Minor discrepancy	No discrepancy
(1) Tooth form	0	1	2
(2) Tooth volume/outline	0	1	2
(3) Color (hue and value)	0	1	2
(4) Surface texture	0	1	2
(5) Translucency	0	1	2
Maximum total Modified PES score			10

### Bone level (B.L.)

The bone level (B.L.) was determined by an intraoral digital radiograph taken parallel to the long axis of the implant, and it was expressed as the mean between the mesial and the distal B.L. To determine the B.L. values, all implants were inserted, leaving the implant neck just at the level of the remaining alveolar bone. After the healing process (three months), and once the prosthesis was inserted, a standardized radiograph was taken using a custom bite block. The distance from the implant neck to the first contact between the bone and the implant was determined. It was measured in both sides, mesial and distal, and the mean between both values was established as the B.L. value. The digital x-ray images were processed and measured with Gesimag Software (Medical Informatics, Barcelona, Spain). At 12-months post-loading, a new B.L. value was measured with the bite block preserved (Table 2).

**Table 2:** Detailed description of the sample description and data obtained, being n: number of patient; Sex: male (M)/female (F); Age: age in years; Teeth: Teeth position; Diam: Implant diameter; Length: Implant length; Osstell 1: osstell measurement at placement; Osstell 2: osstell measurement before placing the definitive prosthesis; Dif: Difference between both osstell measurements; Group: T (treatment)/C (control); BL1 (bone level measurement in mm. at loading); BL2 (bone level measurement in mm. at 12-months post-loading); Dif: Difference between both bone level measurements; PES/WES: PES/WES score obtained; Result: Success (S)/Failure (F).

n	Sex	Age	Teeth	Diam	Length	Osstell1	Osstell2	Dif	Group	BL1	BL2	Dif	PES/WES	Result
1	M	57	12	3.75	10	60	71	11	T	1.64	2	0.36	18	S
2	F	59	12	3.75	10	63	68	5	T	1.08	0.64	− 0.44	16	S
3	F	59	22	3.75	10	68	64	− 4	T	0.51	0.47	− 0.04	14	S
4	F	66	12	3.75	10	63	68	5	T	1.15	1.72	0.57	15	S
5	F	76	21	3.75	10	58	72	14	C	0.45	− 0.65	− 1.1	14	S
6	F	74	12	3.75	10	64	68	4	T	1.6	1.35	− 0.25	14	S
7	F	76	12	3.75	10	64	69	5	C	0.85	1.34	0.49	14	S
8	M	69	21	3.75	10	64	70	6	C	0.15	− 0.6	− 0.75	15	S
9	F	70	22	3.75	10	62	67	5	C	0.25	0.74	0.49	17	S
10	M	55	11	3.75	10	57	65	8	T	− 0.3	0.19	0.49	14	S
11	F	76	12	3.75	10	52	58	6	C	1	− 0.4	− 1.4	14	S
12	F	89	21	3.75	10	48	57	9	C	− 0.75	− 0.27	0.48	17	S
13	F	50	22	3.75	10	57	30	− 27	C	− 0.4	− 1.15	− 0.75	14	F
14	M	55	22	3.75	10	58	65	7	C	0.75	0.9	0.15	14	S
15	M	61	11	3.75	10	68	69	1	C	1.3	0.1	− 1.2	15	S
16	M	59	12	3.75	10	60	65	5	C	0.25	0.74	0.49	18	S
17	F	55	12	3.75	10	61	66	5	T	0.3	− 0.45	− 0.75	14	S
18	M	65	12	3.75	10	62	67	5	T	0.52	1.01	0.49	14	S
19	F	66	11	3.75	10	61	67	6	T	0.57	1.06	0.49	15	S
20	F	67	22	3.75	10	63	68	5	T	0.55	1.04	0.49	16	S
MEAN		65.2				60.65	64.7	4.05		0.5735	0.489	− 0.0845	15.1	

### Operator calibration

The same calibrated single operator measured all the photographs (SMG). All the records were numbered and were randomly reevaluated without prior knowledge of the operator so that the reliability index could be calculated. The intraclass correlation coefficient (ICC) was calculated to consider operator calibration. A value equal or higher of 0.8 was regarded as reliable ICC, being the value 1 equivalent to 100% agreement and the value 0 equivalent to 0% agreement between the different measurements.

### Statistical analysis

The SPSS version 23.0 statistical package (SPSS Inc., Chicago, IL, USA) was used for statistical analysis. The normality of the sample was determined with the Shapiro–Wilk test. Descriptive statistical data were evaluated with the exploratory analyses of the Tukey test. Quantitative mean data (PES/WES, ISQ, and B.L.) were assessed with the nonparametric Wilcoxon-Mann–Whitney U-test to analyze the inferential statistical. Statistical significance was considered at *p* < 0.05.

## Results

### Sample descriptive results

A total of twenty patients (ten control and ten experimental) were recruited, being seven men and thirteen women with a mean age of 65.2 (C.I. 60.6–69.7), ranged from 50 to 89 years old, 35% male and 65% female. The control group was represented by four men and six women with a mean age of 68.1 (C.I. 59.6–76.5), while the experimental group was represented by three men and seven women with an mean age of 62.3 (C.I. 57.8–66.7) (Table 3).

**Table 3:** Baseline patient characteristics by ramdomization arm.

Group	n	Age mean	Age median	Minimum age	Maximum age	Male	Female
Experimental	10	68.1	69.5	50	89	3	7
Control	10	62.3	62	55	74	4	6

Fourteen implants were placed in upper central incisors and six implants in upper lateral incisors. No adverse events were observed during the course of the study. There was one failure in the control group at six months due to mobility in a 50 years old woman, although without inflammation or pain. This implant was removed, and the patient dropout the study. All teeth had a previous canal root treatment with no active apical focus and were extracted due to unrecoverable crown fracture.

### Intraclass correlation coefficient (ICC)

The ICC obtained for the PES was 0.954, for the WES was 0.625 and for PES/WES was 0.93.

### ISQ Values

#### ISQ Values during implant insertion. Primary stability

The descriptive analysis of the ISQ values obtained during implant insertion was a mean of 59.1 (C.I. 54.8–63.3) for the control group and 62.2 (C.I. 60.1–64.2) for the experimental group. No statistically significant differences were shown (*p* = 0.280) (Table 4), although the experimental group showed less dispersion and a lightly higher value.

**Table 4:** Summary statistics by ramdomization arm and *p*-values for ISQ values (at baseline and 3-months), PES/WES values at 12-months, BL values (at 3-months and 12-months).

Group	n	ISQ basal	ISQ 3-months	PES/WES 12-months	BL 3-months	BL 12-months
Experimental	10	62.2	67.2	15	0.38	0.9
Control	10	59.1	62.2	15.2	0.76	0.75
*p* value		0.28	0.684	0.912	0.165	0.035

#### ISQ Values at three months. Secondary stability

**Figure 5:**
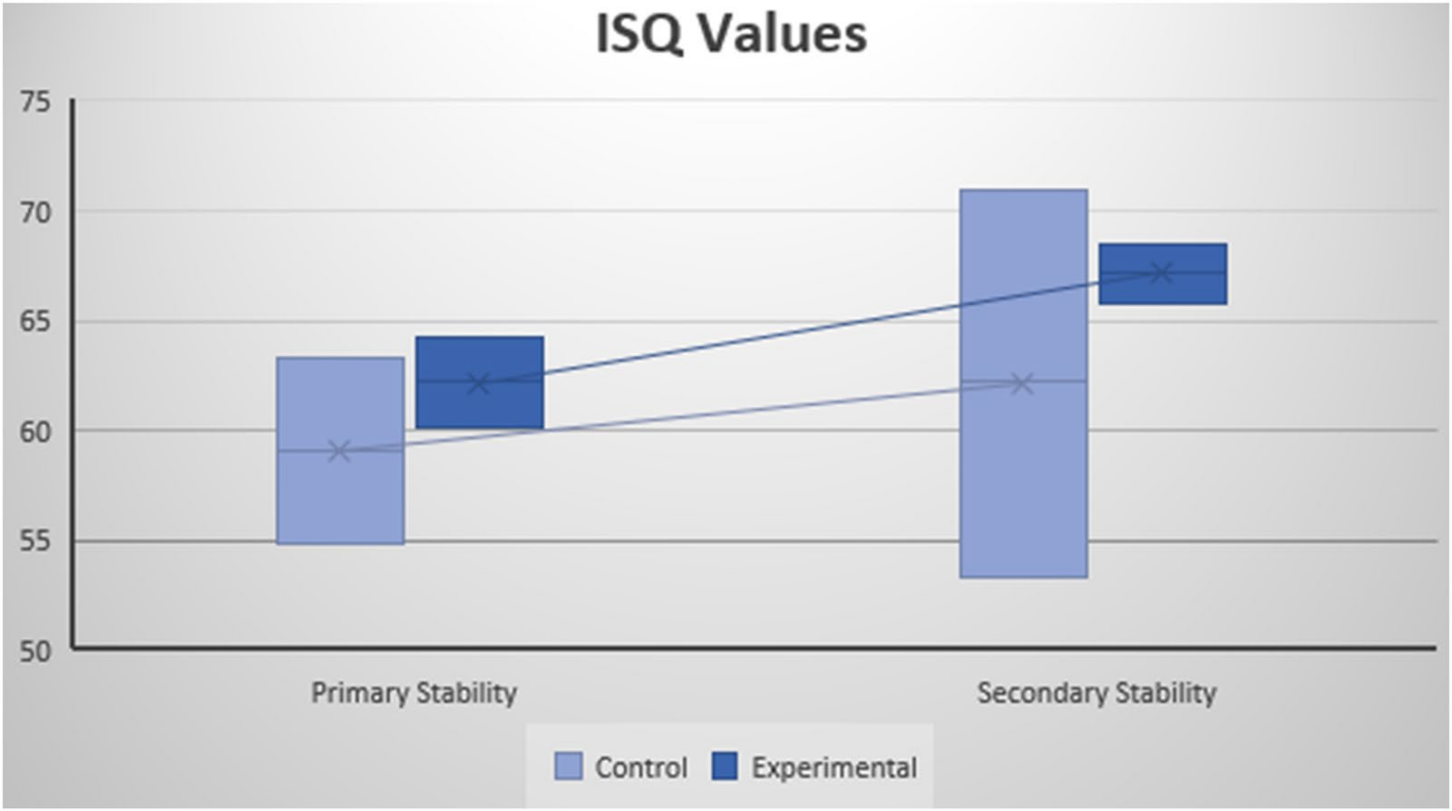
Mean and C.I. of ISQ values for the control group and experimental group during implant insertion (primary stability) and at three months after implant insertion (secondary stability).

The mean ISQ value for the control group was 62.2 (C.I. 53.3–71.0), and for the experimental group was 67.2 (C.I. 65.8–68.5). Again, no statistically significant differences were shown (*p* = 0.684) (Table 4), but the experimental group showed less dispersion and a higher value (Fig. 5).

### PES/WES values

PES/WES was evaluated at twelve months post-loading. The mean value in the control group was 15 (C.I. 12.68—17.32), and in the experimental group was 15.20 (C.I. 11.99—18.41). No significant differences were shown (*p* = 0.912) (Table 4).

### B.L. Values

#### B.L. Values at three months after surgery

**Figure 6:**
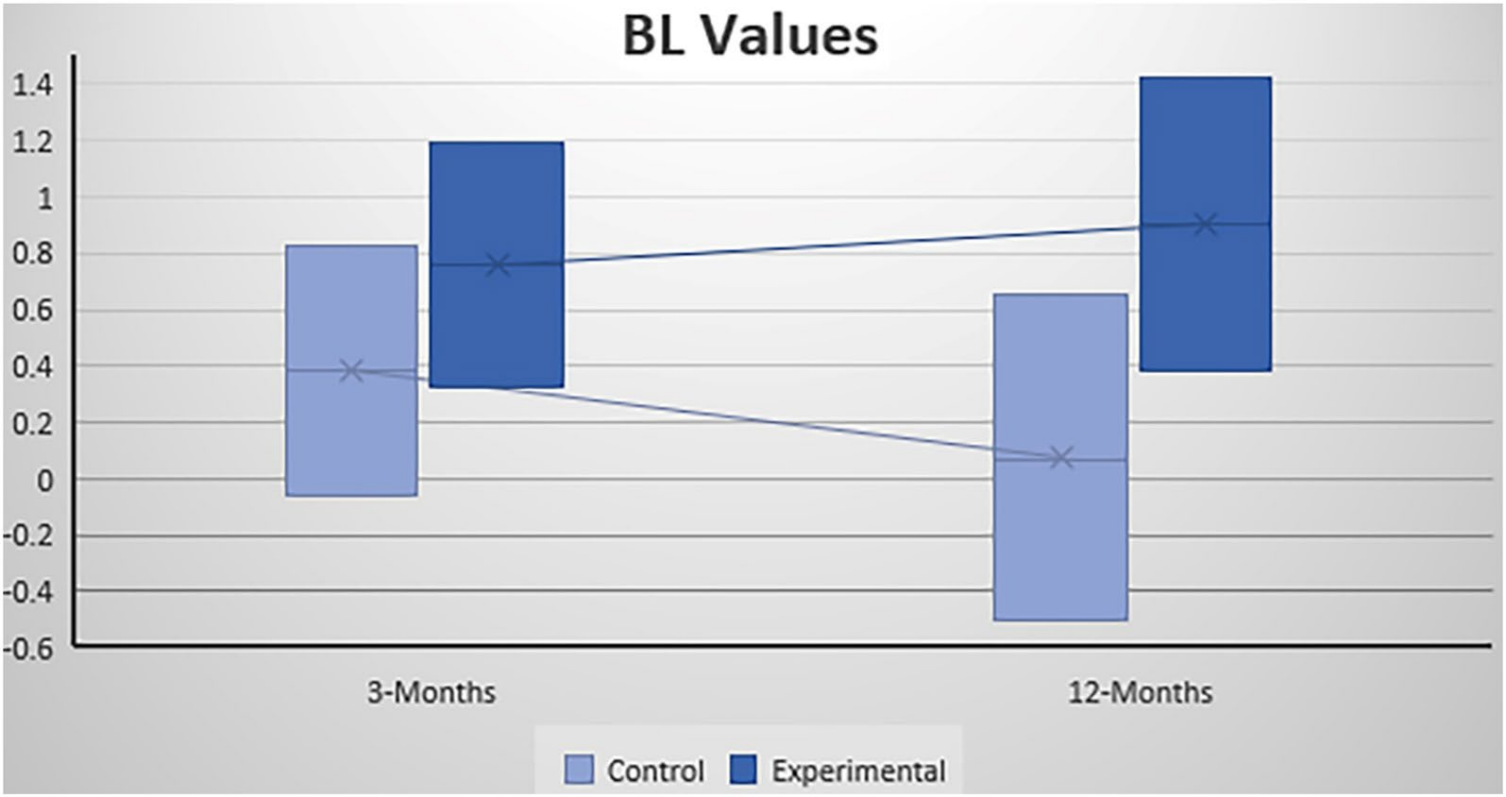
Mean and C.I. for BL values for the control group and experimental group at three months after implant placement and at twelve months after loading.

For the control group, the mean B.L. value was 0.38 mm (C.I. − 0.06 to + 0.83); meanwhile, for the experimental group, the mean BL value was 0.76 mm (C.I. 0.33–1.19) (Fig. 6). There were no statistically significant differences (*p* = 0.165) (Table 4).

#### BL Values at twelve months post-loading

The control group showed a mean BL value of 0.07 mm (C.I. − 0.50 to  + 0.65), while the experimental group showed a mean BL value of 0.90 mm (C.I. 0.38–1.42) (Fig. 6). There were no significant differences (*p* = 0.035) (Table 4).

## Discussion

After the publication of the first pioneer studies in the field of immediate implantology, some publications have appeared showing a particular interest^22–24^. In these studies, the authors concluded that the placement of immediate implants does not prevent crestal bone reabsorption both vertically and horizontally. Our results yield the same conclusions, as in most cases, there was a loss of bone level.

Many researchers have tried to determine the success rate of immediate implants related to delayed implants, which have been considered the gold standard. Del Fabro et al. showed a survival rate of 95% for immediate implants, comparable to the delayed implants^25^. In our study, we had one implant failed at six months in the control group, presenting a success rate of 95%, being this data in agreement with the referred author.

In our study, only those patients with grade 1 in the classification of Ellian et al. were selected^26^. Among these pre-selected patients, those patients who also had a rating 1, 2, or 3 in the Kan’s classification^27^ were selected. These strict choice criteria made our study candidates very favorable. This rigorous selection meant that only 10% of the candidates were selected to participate. We strongly believe that this selection has been part of the success and high stability achieved, but at the same time, it limits the extrapolation of the results to the whole population.

We determined the three-dimensional position of the implant by the prosthetically guided planning, and the results were also improved by the use of the platform switching and the placement of the implants at bone level^28^.

We did not use any bone or soft tissue graft, only patients with a thick phenotype were selected. To determine the patient´s suitability, we relieve the transparency criteria of the periodontal probe^29^. This selection involved an adequate width and height of the keratinized mucosa.

As for bone grafting, when we place immediate implants, it is generally accepted that the space between the vestibular cortical and the implant (GAP) exceeding two mm should be grafted with bone substitutes^30^, but not all authors agree with this statement^31,32^, being one of the topics without conclusive results at present^16,17^. In our study, we decided not to perform any bone graft in the GAP regardless of the distance between the vestibular cortical and the implant.

We intended to evaluate only the effect that the implant design could have on the aesthetics and stability of the implant. Any other variable would have meant some degree of confusion. For this reason, the selection according to the criteria of Ellian et al. and Kan et al. was so restrictive.

Both implant designs achieved satisfactory clinical results. Only one control group implant was lost, which is generally within expectations. It should be noted that the exhaustive selection of patients created the appropriate clinical framework for these results. This is evident from the absence of statistically significant differences between the two implant designs. However, we believe that these results could have favored the experimental group in clinical situations with low-density bone or alveoli type 2, because the new implant of the experimental group has a different thread design that exert more significant pressure on the cortical, favoring the appearance of small bone resorption that, however, does not affect the aesthetic score in the present study.

It must be noted that the implant surface design (outer shapes and thread pitches) influence directly in the resonance frequency values, as well as the implant shape, being tapered implants the most sensitives to bone maturation levels^33^.

One of the limitations to keep in mind is that there are authors who claim that one single value, such as the ISQ, is not suitable to state the level of osseointegration since it is the result of several factors such as cortical bone thickness, trabecular bone properties or bone to implant contact area^34^.

As for secondary stability (at three months after implant placement), the excellent response of RBM surfaces is already known. We did not expect to find differences as it occurred. Once osseointegration has been achieved, the similarities of the intrinsic characteristics of both implants are probably not a significant difference in BIC values, as demonstrated in animal studies^35^.Regarding BL values, both systems have managed to maintain the alveolar bone without the use of graft materials. It shows some interest that three implants had bone levels that had exceeded the implant shoulder at three months (two controls and one experimental). In the twelve-months follow-up, we also did not find significant differences between the two implant designs, and six implants showed bone formation above the implant shoulder (five controls and one experimental).

The PES/WES values for both systems resulted in adequate aesthetic indexes, without significant differences. All values improved with the time progression except for the vestibular contour. Our results indicate a gradual collapse of the gingival contour with no repercussions as to the other variables of the PES. Both the papillae, the margin, the color and texture of the soft tissue improved over time. These findings could provide indirect evidence in favor of grafting with bone substitutes in the GAP when an immediate implant is placed. The WES index remained unchanged as expected throughout the follow-up period.

Our study possesses some limitations, such as those already described regarding exquisite patient selection and the inclusion and exclusion criteria. Due to the small sample size and power, we present this study as a pilot clinical double-blind randomized study. A higher sample size would be desirable for future research. Thus, we only present the results of a twelve-months follow-up after loading, and we offer only clinical evaluation but not histological determination since the study was done in vivo and humans. But the study also has some strengths such as the randomized distribution of the sample, the blind evaluation, the management in a controlled environment, and the non-use of bone graft or soft tissue graft in any patient, which could disturb the results. There was no external source of financing, and none of the authors received any financial or material retribution. Our results could be of particular interest to the clinic that handles this clinical situation day by day. The clinical relevance of this study is that the diagnosis and proper treatment planning must be made to choose the right patient for immediate implants to achieve the best clinical results.

## Conclusions

Within the limitations of this study, we can conclude that:

- Both implant designs can induce osseointegration under immediate placement.
- Both implant designs offer an acceptable value of ISQ for primary and secondary stability.
- Proper stable radiographic crestal bone levels are maintained in both systems at 12-months follow-up.
- Both systems showed similar PES/WES values.
- The difference in design for both implant systems does not affect primary/secondary stability, bone level, or PES/WES value when the patient is appropriately selected for immediate implant placement.
